# Effectiveness of Digital Guided Self-help Mindfulness Training During Pregnancy on Maternal Psychological Distress and Infant Neuropsychological Development: Randomized Controlled Trial

**DOI:** 10.2196/41298

**Published:** 2023-02-10

**Authors:** Xuan Zhang, Yang Li, Juan Wang, Fangxiang Mao, Liuliu Wu, Yongqi Huang, Jiwei Sun, Fenglin Cao

**Affiliations:** 1 School of Nursing and Rehabilitation Shandong University Jinan China; 2 Austin School of Nursing The University of Texas Texas, TX United States; 3 Cheeloo College of Medicine Shandong University Jinan China

**Keywords:** digital, mobile health, mHealth, guided self-help, psychological distress, pregnancy, psychosocial intervention, mindfulness, infant, neuropsychological performance

## Abstract

**Background:**

Maternal psychological distress during pregnancy is associated with unfavorable outcomes in infants. Mindfulness-based interventions (MBIs) can effectively alleviate psychological distress, but there are often barriers to the access of face-to-face interventions.

**Objective:**

This study aimed to investigate the effectiveness of a digital guided self-help (GSH) MBI (GSH-MBI) in reducing maternal psychological distress and improving infant neuropsychological performance.

**Methods:**

This was a randomized controlled trial. We recruited 160 women who were 12 to 20 weeks pregnant and exhibited psychological distress. We randomized them into a digital GSH-MBI group and a control group (usual perinatal care). The digital GSH-MBI consisted of a 6-week intervention through a WeChat mini program, with a daily reminder sent to the participants by a research assistant via WeChat. The primary outcomes consisted of maternal psychological distress, including depression, anxiety, and pregnancy-related anxiety symptoms, which were assessed at 6 time points from baseline to 6 months post partum (only pregnancy-related anxiety symptoms were assessed 3 times during pregnancy). The secondary outcomes were infant neuropsychological outcomes, including temperament and developmental behaviors, which were assessed at 6 weeks and 6 months post partum.

**Results:**

Compared with the control group, the digital GSH-MBI group showed a significant reduction in depression, anxiety, and pregnancy-related anxiety symptoms. In addition, the scores of the digital GSH-MBI group were lower than those of the control group for the 3 types of infant temperament at 6 weeks post partum, including quality of mood, distractibility, and adaptability.

**Conclusions:**

Digital GSH-MBIs are effective in alleviating psychological distress among pregnant women and protecting infant outcomes.

**Trial Registration:**

Chinese Clinical Trial Register ChiCTR2000040717; https://www.chictr.org.cn/showproj.aspx?proj=65376

## Introduction

Psychological distress during pregnancy is a common condition worldwide. The overall prevalence of depression and anxiety symptoms during pregnancy is 22.4% and 22.9%, respectively [1,2]. Psychological distress during pregnancy can have devastating effects such as maternal postpartum depression and adverse neonatal outcomes such as premature delivery, low birth weight, and intrauterine growth restriction [3]. In addition, according to the Developmental Origins of Health and Disease hypothesis [4], maternal psychological distress during pregnancy may also affect the fetus through the developmental process, permanently altering the structure and function of the organ and the biofeedback system, thereby affecting the infant’s neurodevelopment. Hence, the design and implementation of psychological interventions to decrease psychological distress during pregnancy and to enhance maternal and infant well-being should be a public health priority.

Mindfulness-based interventions (MBIs) teach participants to recognize habitual patterns of response, to be more mindful in their daily lives, and to make changes in how they relate to their thoughts, feelings, bodily sensations, and external environments [5]. Previous studies have preliminarily identified the effectiveness of MBIs during pregnancy [6,7]; however, most of these studies were nonrandomized controlled pilot trials or had high dropout rates and poor compliance. More robust evidence in future research is necessary [8]. In addition, pregnant women frequently encounter practical and psychosocial barriers to accessing face-to-face psychological interventions, especially in low- and middle-income countries [9]. These barriers include a lack of professional staff who can facilitate psychological interventions, cost concerns, and stigma related to mental disorders [10,11].

The digital guided self-help (GSH) approach is an innovative approach that circumvents many barriers to disseminating face-to-face interventions. It allows patients to take home a standardized psychological intervention protocol (downloaded from the internet) that functions more or less independently [12,13]. Professionals and paraprofessionals can make minimal contact with participants in the intervention process, playing a positive role in guidance [12,14]. The digital GSH approach has rapidly emerged as a viable alternative or addition to face-to-face interventions [15], especially during the COVID-19 pandemic [16]. The application of digital GSH-MBIs in pregnancy is still in its infancy. Two preliminary randomized controlled trials (RCTs) involving pregnant women used digital MBIs. Of them, one was a pilot study [17] and the other had a very low completion rate [18].

In recent years, we have developed 3 generations of a digital GSH-MBI that target psychological distress during pregnancy. The first generation [19] involved WeChat as the intervention medium. With a small sample size and high dropout rate, we did not find effectiveness in reducing depression and anxiety in pregnant women. To increase the retention rate, the second-generation MBI [9] applied a smartphone app as the intervention medium. However, there were still a high dropout rate and low completion rate, which may explain the small effect size detected only immediately after the intervention.

This study includes the third-generation digital GSH-MBI. On the basis of our prior research, we made further improvements. First, the intervention program was presented in video and audio formats, as past studies have shown that psychological education produces better intervention effects in a video format than in a text format [20]. The first 2 generations of the GSH-MBI program were presented only in text and audio formats. Second, we created a mini program grounded in WeChat named “Take it easy, sunny mood” as an intervention medium. WeChat (Tencent) is the most popular smartphone app in China and is regarded as one of the leading social networks worldwide [21]; it has 902 million daily users. Participants do not need to log on to a specific website or download a new app to access the intervention. Ease of access to information might increase participation and compliance. Thus, our first aim was to examine the effect of the digital GSH-MBI based on a WeChat mini program on reducing psychological distress (ie, depression, anxiety, and pregnancy-related anxiety symptoms) among pregnant women, with a relatively large sample size and an approximately 1-year follow-up.

Although the impact of psychological distress during pregnancy on infant neurodevelopmental outcomes was discovered years ago, little progress has been made in addressing this problem [22]. Few studies have investigated the effects of maternal face-to-face psychological interventions on child neuropsychological development. Of the 3 RCTs that investigated the ripple effect of cognitive-behavioral therapy during pregnancy on child outcomes, 2 [22,23] had small sample sizes (N=29 and N=25) and the third trial [24] found no beneficial effects on maternal psychological distress and child outcomes. In addition, the “Thinking Healthy Program” [25], which was a perinatal psychological intervention, showed a very limited effect on child neurodevelopment. However, the intervention program focused mainly on the postpartum period. Therefore, it is unclear whether psychological interventions can affect infant neuropsychological development by ameliorating maternal psychological distress during pregnancy, and more well-designed RCTs are needed. To the best of our knowledge, no study has examined the ripple effect of digital psychological interventions that target psychological distress among pregnant women on infant neuropsychological development. Thus, our second aim was to conduct an exploratory analysis to examine the ripple effect of our digital GSH-MBI on infant neuropsychological development. If the ripple effect existed, we would further analyze whether the ripple effect was correlated with reduced depression, anxiety, or pregnancy-related anxiety symptoms in pregnant women.

## Methods

### Ethics Approval

The study protocol was reviewed and approved by the Ethics Committee of Shandong University School of Nursing (2020-R-025). Informed consent was obtained from all participants to participate in the study. The authors assert that all procedures contributing to this work comply with the ethical standards of the relevant national and institutional committees on human experimentation and the Helsinki Declaration of 1975, as revised in 2008.

### Study Design and Participant Eligibility

This study was a 2-arm RCT. The participants were pregnant women who underwent routine obstetric examinations at the outpatient centers of 2 comprehensive tertiary hospitals in Shandong Province from December 2020 to April 2021. The inclusion criteria were as follows: the participant must be aged ≥18 years, have single pregnancy, be at 12 to 20 weeks of gestation, have Edinburgh Postnatal Depression Scale (EPDS) score of ≥9 or Generalized Anxiety Disorder 7-item (GAD-7) scale score of ≥5, be fluent in reading and writing Chinese, not participated in any other psychological intervention, and be able to access the WeChat mini program. We excluded participants if they had suicidal ideation (score of item 10 of EPDS ≥1), serious mental disorders (eg, schizophrenia and bipolar disorder), physical illnesses (eg, cancer and cardiovascular or cerebrovascular diseases), drug abuse or dependence, or prior experience in mindfulness exercises. This study was registered in the Chinese Clinical Trial Register (identifier: ChiCTR2000040717).

### Sample Size

We used the G* Power [26] software to calculate the sample size. As reported in previous studies, mindfulness intervention among pregnant women revealed a medium effect size for anxiety and depression [6], and the effect sizes of self-help intervention for depression and anxiety were small to medium [27]. On the basis of a medium intervention effect (Cohen *d*=0.50), an estimated total sample size of 128 was needed to achieve 80% power and 2-sided *P*<.05. Considering a 20% attrition rate, 160 participants were included in the study.

### Procedures

We invited pregnant women at 12 to 20 weeks of gestation (baseline, T1) to participate in screening for symptoms of anxiety and depression during routine obstetric visits. Then, we invited eligible women to complete a demographic questionnaire and pregnancy-related anxiety scale. Random sequences were generated using a computerized random number generator. Random numbers were placed in opaque envelopes and assigned to participants according to the sequence of participant enrollment. Eligible participants who agreed to participate were randomized 1:1 to one of 2 groups: the digital GSH-MBI group (ie, usual perinatal care and mindfulness intervention) or the control group (ie, usual perinatal care) according to the random numbers in the envelopes. Data collectors were blinded to the treatment allocation. Women in the digital GSH-MBI group were told about the requirements and notices of the intervention and then started receiving the mindfulness intervention.

### The Intervention: Mindfulness

**Figure 1:**
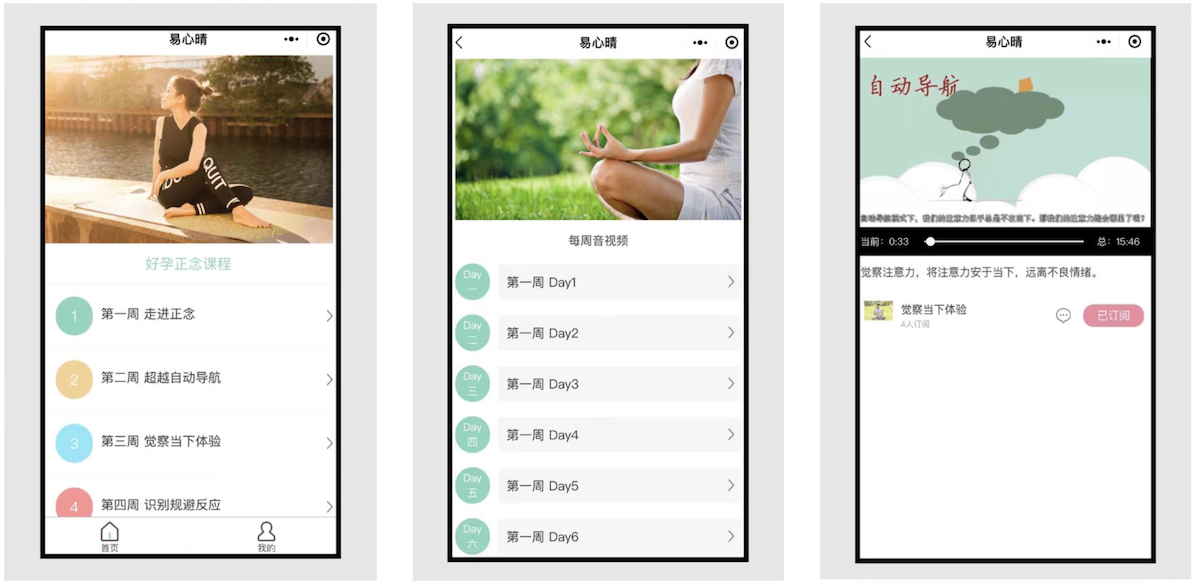
Screenshot of WeChat mini program.

The intervention program included 6 modules, each of which lasted for 1 week and was delivered on a WeChat mini program. Each module consisted of thematic lessons and homework. On the first day of each week, participants viewed animated videos that provided thematic lessons of each module. Each video was 10- to 20-minute long and included a variety of cartoon images and mindfulness practice demonstrations recorded by our research team. We hoped that this would increase the participants’ interest in the course content and improve their compliance. For the remaining 6 days of each week, participants were asked to do homework, including formal and informal practices. The formal practice involved daily audio-based practices, such as mindful breathing and body scan. Participants were also encouraged to engage in informal practice daily, such as mindfulness in everyday life or 3-minute breathing space exercises. Participants in the intervention group were sent standardized practice reminders every day by the research assistant via WeChat. The content of the reminder was, “Ding ~ remember to attend class! Today is week X, day X.” Multimedia Appendix 1 illustrates the program outline. Figure 1 shows a screenshot of the mini program.

### Data Collection

#### Outcomes

The primary outcome was maternal psychological distress, including symptoms of depression, anxiety, and pregnancy-related anxiety. We assessed depression and anxiety using the EPDS [28] and GAD-7 [29], respectively, at baseline (12-20 weeks of gestation, T1); immediately after the intervention (approximately 20-28 weeks of gestation, T2); before birth (36-37 weeks of gestation, T3); and at 6 weeks (T4), 3 months (T5), and 6 months post partum (T6). Symptoms of pregnancy-related anxiety refer to symptoms specific to pregnancy, including concerns about the health of the baby and oneself, as well as concerns about childbirth [30]. We assessed the symptoms of pregnancy-related anxiety at T1, T2, and T3 using the pregnancy-related anxiety scale [30].

The EPDS comprises 10 items, with responses ranging from 0 to 3. Higher total scores indicate more depressive symptoms. An EPDS score of 9 is recommended for screening for depression in postpartum Chinese women [28] and has been widely used among pregnant women in China [19,31]. A score of ≥1 on the EPDS item-10 (“I have thought of hurting myself”) indicates the presence of suicide ideation [32,33]. The GAD-7 contains 7 items rated from 0 to 3. The summed score is calculated to determine the severity of the anxiety symptoms. A GAD-7 score of ≥5 indicates the presence of anxiety [29], and this tool has also been widely used among pregnant women in China [19]. The pregnancy-related anxiety scale comprises 13 items ranging from 1 to 4 [30]. Higher EPDS, GAD-7, and pregnancy-related anxiety scale scores indicate more severe psychological distress [30].

The secondary outcomes were infant neuropsychological development, including temperament and developmental behaviors. We assessed infant temperament at 6 weeks post partum using the Early Infancy Temperament Questionnaire [34,35], which is suitable for infants aged 1 to 4 months. It consists of 76 items (with responses ranging from 1 to 6) and 9 dimensions, including activity level, rhythmicity, approach, intensity of reaction, quality of mood, attention span, distractibility, threshold of reaction, and adaptability. Higher scores reflect more activities, worse rhythm, easier withdrawal, stronger reaction, more negative mood, less persistence, less distraction, lower threshold of reaction, and slower adaptation. We assessed infant temperament at 6 months post partum using the Very Short Form of the Infant Behavior Questionnaire–Revised [36], which is suitable for infants aged >3 months. It consists of 3 dimensions: surgency, negative affect, and effortful control. Higher summed scores indicate higher levels of particular temperament characteristics. We measured infant developmental behaviors at 6 weeks and 6 months post partum using the Age and Stages Questionnaire-Third Edition [37]. The Age and Stages Questionnaire-Third Edition consists of 30 items rated on a 3-point Likert scale, with a total score ranging between 0 and 60 for each domain. It assesses the development of 5 functional areas in infants: communication, gross motor, fine motor, problem-solving, and personal-social, with higher scores indicating better developmental behaviors. Both infant temperament and developmental behaviors were rated by the mothers.

#### Demographic Characteristics, Pregnancy Conditions, and Neonatal Outcomes

We collected baseline data on the participants’ demographic characteristics and pregnancy-related conditions using questions developed specifically for this study. These questions asked about age, nationality (Han or National minority), education (below undergraduate or undergraduate or above), marital status (married or single), average monthly household income (<6000 yuan or ≥6000 yuan, CNY 6000 yuan [US $884]), residence (urban or rural), height, gestational age, prepregnancy weight, pregnancy weight, parity (primipara or multipara), and complications during pregnancy (yes or no). We acquired information on neonatal outcomes, including birth weight and gestational age at delivery, from the hospital registry databases.

#### Adherence

We defined adherence as the number of weeks of completed modules, and we defined module completion as at least 4 days of formal practice per week. We defined intervention completion as completion of all 6 modules. The backend system of the WeChat mini program could record the participants’ formal practice time each day. We calculated the intervention completion rate as the proportion of participants who completed the intervention divided by the number of participants who received the intervention.

### Statistical Analysis

We used the Little test to assess whether the missing mechanism is completely random. Because the *P* value for the Little test was not significant, we did not reject the null hypothesis that data were missing completely at random. Missing completely at random means that the subsample consisting of participants with complete (or nonmissing) data is representative of the overall sample [38]. We examined baseline differences between the digital GSH-MBI and control groups using descriptive information per the recommendations in the CONSORT (Consolidated Standards of Reporting Trials) guidelines.

We used the generalized estimated equation with an unstructured working correlation matrix to explore the effect of the digital GSH-MBI on maternal psychological distress, including symptoms of depression, anxiety, and pregnancy-related anxiety. The generalized estimated equation does not require imputing missing values, which is especially advantageous in longitudinal studies with missing data, as all available data are used and no cases are deleted. We followed the intention-to-treat analysis and performed a sensitivity analysis using a mixed-effects modeling approach (Multimedia Appendix 2) that assumed that data were missing at random.

We used linear regression to scrutinize the effects of the intervention on infant neuropsychological development. Infant sex, maternal age, maternal education, and average monthly household income were included as covariates in the regression analysis according to previous RCT studies that focused on maternal psychological intervention and child neurodevelopment [25]. This part of the analysis was a complete-case analysis. Owing to multiple comparisons, we used the false discovery rate with the Benjamini-Hochberg correction to adjust the *P* values.

We performed further analyses using Amos (version 26.0; IBM Corp) to examine the effects of the intervention on infant neuropsychological development by mitigating maternal psychological distress. The model of mediation was established with an independent variable (X, treatment allocation), a mediating variable (M, maternal psychological distress at T2 or T3), and a dependent variable (Y, infant outcomes). We performed a partial correlation analysis to investigate the relationship between maternal psychological distress at T2 or T3 and infant neuropsychological development after adjusting for psychological distress at T1. We included only maternal psychological distress variables that were significantly correlated with infant neuropsychological development in the mediation analysis.

We conducted statistical analyses using SPSS (version 26.0; IBM Corp). We calculated Cohen *d* as the effect size of the intervention on the maternal and infant outcomes. We performed all tests using an α level of .05 (2-sided) and 95% CIs.

## Results

### Overview

**Figure 2:**
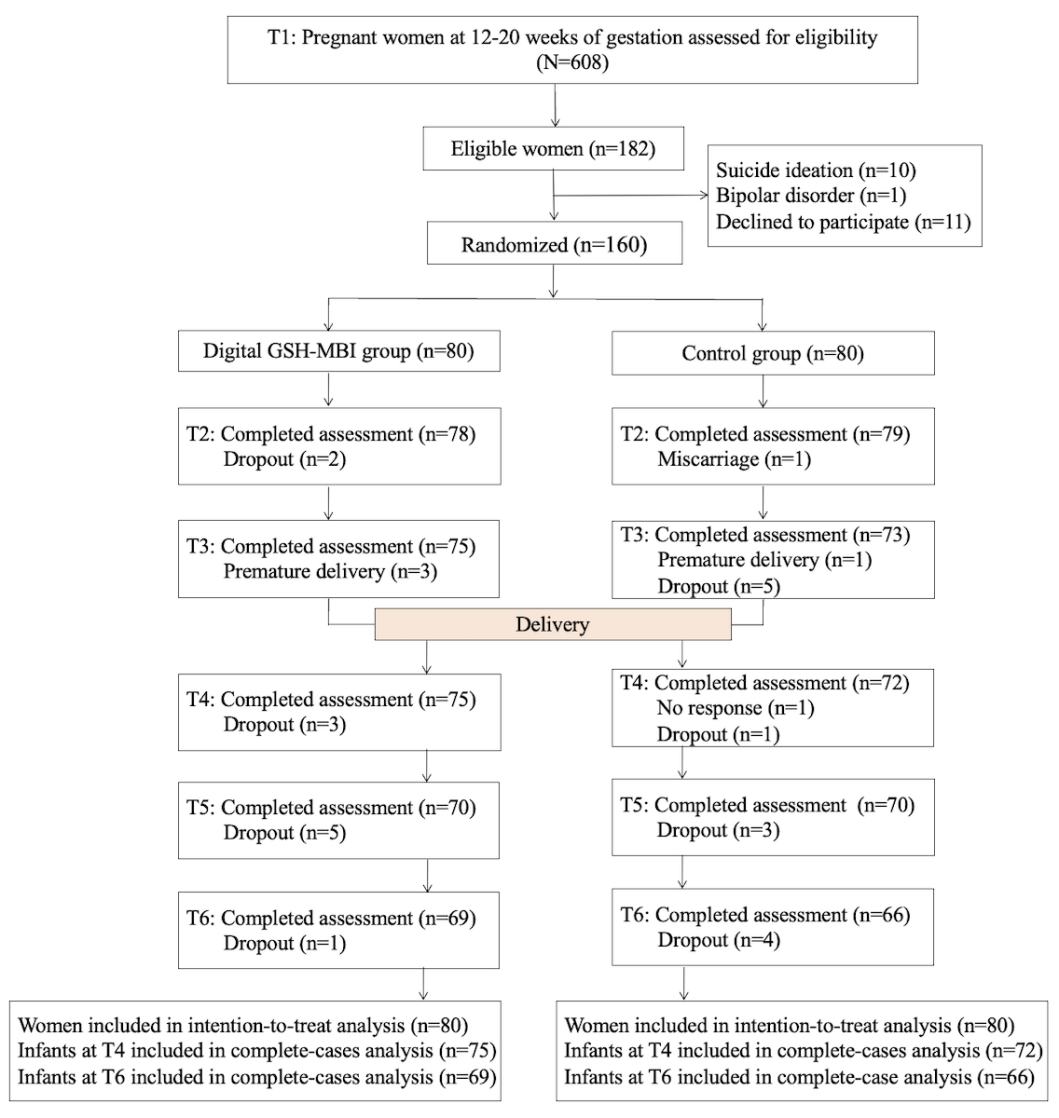
Participant flow diagram. GSH: guided self-help; MBI: mindfulness-based intervention. T1: baseline (12-20 weeks of gestation); T2: immediately after the intervention (approximately 20-28 weeks of gestation); T3: before birth (36-37 weeks of gestation); T4: 6 weeks post partum; T5: 3 months post partum; T6: 6 months post partum.

We approached and screened 608 pregnant women at 12 to 20 weeks of gestation for eligibility, and we obtained a final sample of 160 (Figure 2). Among the 160 participants, 135 (84.4%) completed the assessment at 6 months post partum; 11 (6.9%) participants dropped out from the intervention group and 14 (8.8%) from the control group (dropout rate: 25/160, 15.6%). Table 1 presents the sample characteristics. Their mean age was 30 (SD 4.29) years, and the mean gestational age was approximately 17 weeks. The infant birth weight was 3375.77 (445.74) grams, and the gestational age at delivery was 38.34 (1.44) weeks. There were no significant differences in birth weight (mean 3305.36, SD 511.90 g vs mean 3447.21, SD 356.45 g; *t*_135_=–1.88; *P*=.06) and gestational age (mean 38.27, SD 1.96 weeks vs mean 38.4, SD 1.14 weeks; *t*_138_=–0.57; *P*=.57) at delivery between the digital GSH-MBI and control groups.

**Table 1:** Demographic characteristics and pregnancy-related conditions of pregnant women.

					Total sample (N=160)		Digital GSH-MBI^a^ group (n=80)		Control group (n=80)
**Demographic characteristics**									
	Age (years), mean (SD)				30.29 (4.29)		30.36 (4.65)		30.21 (3.93)
	**Nationality, n (%)**								
		Han		158 (98.8)		79 (98.8)		79 (98.8)	
		National minority		2 (1.2)		1 (1.2)		1 (1.2)	
	**Education, n (%)**								
		Below undergraduate		62 (38.8)		28 (35.0)		34 (42.5)	
		Undergraduate or above		98 (61.2)		52 (65.0)		46 (57.5)	
	**Marriage, n (%)**								
		Married		156 (97.5)		77 (96.2)		79 (98.8)	
		Single		4 (2.5)		3 (3.8)		1 (1.2)	
	**Average monthly household income (Yuan; CNY 1 yuan=US $6.8), n (%)**								
		＜6000		43 (26.9)		22 (27.5)		21 (26.2)	
		≥6000		117 (73.1)		58 (72.5)		59 (73.8)	
	**Residence, n (%)**								
		Urban		141 (88.1)		71 (88.8)		70 (87.5)	
		Rural		19 (11.9)		9 (11.2)		10 (12.5)	
**Pregnancy-related conditions, mean (SD)**									
	Gestational age (weeks)				16.69 (1.60)		16.71 (1.67)		16.66 (1.53)
	Prepregnancy BMI (kg/m^2)^				21.64 (3.27)		21.14 (3.11)		22.14 (3.36)
	Pregnancy BMI (kg/m^2^)				22.64 (3.49)		22.21 (3.30)		23.08 (3.66)
	**Parity, n (%)**								
			Primipara	94 (58.8)		45 (56.2)		49 (61.2)	
			Multipara	66 (41.2)		35 (43.8)		31 (38.8)	
	**Complications during pregnancy, n (%)**								
			Yes	43 (27.5)		19 (23.8)		24 (30.4)	
			No	116 (72.5)		61 (76.2)		55 (69.6)	

^a^GSH-MBI: guided self-help mindfulness-based intervention.

### Maternal Psychological Distress

**Figure 3:**
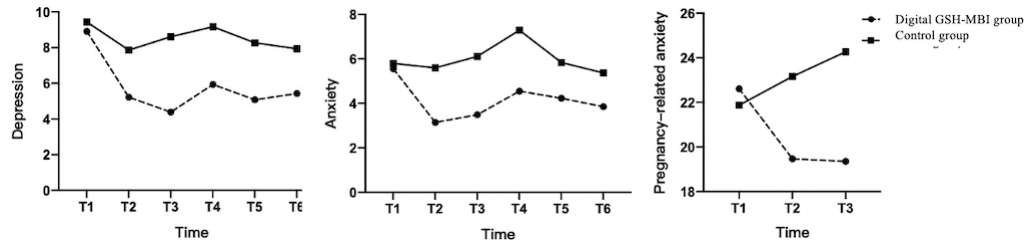
Changes in maternal psychological distress over time in the digital guided self-help (GSH)–mindfulness-based intervention (MBI) group and the control group. T1: baseline (12-20 weeks of gestation); T2: immediately after the intervention (approximately 20-28 weeks of gestation); T3: before birth (36-37 weeks of gestation); T4: 6 weeks post partum; T5: 3 months post partum; T6: 6 months post partum.

As shown in Figure 3 and Table 2, the results indicate a significant time × group interaction for symptoms of depression (Wald *χ*^2^_5_=20.6; *P*=.001), anxiety (Wald *χ*^2^_5_=24.7; *P<*.001), and pregnancy-related anxiety (Wald *χ*^2^_2_=46.5; *P<*.001). Changes in depression, anxiety, and pregnancy-related anxiety symptoms in the postintervention period differed significantly between the digital GSH-MBI and control groups. In the digital GSH-MBI group, depression symptoms that were observed from T2 to T6 were all significantly lower than those observed in the control group, with Cohen *d* values of 0.56, 0.84, 0.59, 0.56, and 0.49, respectively. Anxiety symptoms observed in the digital GSH-MBI group from T2 to T6 were all significantly lower than those observed in the control group, with Cohen *d* of 0.85, 0.82, 0.69, 0.39, and 0.53, respectively. Pregnancy-related anxiety symptoms in the digital GSH-MBI group at T2 and T3 were significantly lower than in the control group, with a Cohen *d* of 0.76 and 0.89, respectively.

**Table 2:** Overall global test results and between-group differences in maternal psychological distress in the generalized estimating equations analysis.

Outcomes and time			Digital GSH-MBI^a^ group, mean (SD)		Control group, mean (SD)		Estimated mean difference, mean (95% CI)		*P* value		Cohen *d*		Group × time				
													Wald *χ*^2^ (*df*)			*P* value	
**Depression**														20.6 (5)			.001
	T1^b^	8.91 (3.54)		9.43 (3.26)		−0.53 (−1.57 to 0.52)		.33		N/A^c^							
	T2^d^	5.21 (4.46)		7.86 (5.07)		−2.64 (−4.12 to −1.16)		＜.001		0.56 (0.24 to 0.87)							
	T3^e^	4.48 (4.22)		8.60 (5.58)		−4.22 (−5.79 to −2.66)		＜.001		0.84 (0.50 to 1.17)							
	T4^f^	5.81 (5.27)		9.25 (6.34)		−3.23 (−5.10 to −1.37)		.001		0.59 (0.26 to 0.92)							
	T5^g^	5.25 (4.47)		8.27 (6.31)		−3.18 (−4.94 to −1.43)		＜.001		0.56 (0.23 to 0.90)							
	T6^h^	5.54 (5.44)		8.45 (6.53)		−2.51 (−4.57 to −0.45)		.02		0.49 (0.15 to 0.83)							
**Anxiety**														24.7 (5)			＜.001
	T1	5.56 (2.61)		5.80 (3.14)		−0.24 (−1.13 to 0.65)		.60		N/A							
	T2	3.14 (2.74)		5.61 (3.04)		−2.46 (−3.36 to −1.56)		＜.001		0.85 (0.52 to 1.18)							
	T3	3.32 (3.19)		6.18 (3.83)		−2.62 (−3.76 to −1.48)		＜.001		0.82 (0.48 to 1.15)							
	T4	4.49 (3.63)		7.31 (4.49)		−2.74 (−4.04 to −1.44)		＜.001		0.69 (0.36 to 1.02)							
	T5	4.34 (3.31)		5.90 (4.71)		−1.60 (−2.91 to −0.29)		.02		0.39 (0.05 to 0.72)							
	T6	3.75 (3.28)		5.90 (4.76)		−1.52 (−2.92 to −0.13)		.03		0.53 (0.19 to 0.87)							
**Pregnancy-related anxiety**														46.5 (2)			＜.001
	T1	22.61 (4.53)		21.88 (4.64)		0.74 (−0.67 to 2.15)		.31		N/A							
	T2	19.47 (4.03)		23.15 (5.55)		−3.70 (−5.20 to −2.19)		＜.001		0.76 (0.43 to 1.08)							
	T3	19.41 (4.98)		24.22 (5.77)		−4.92 (−6.60 to −3.23)		＜.001		0.89 (0.55 to 1.23)							

^a^GSH-MBI: guided self-help mindfulness-based intervention.

^b^T1: baseline (12-20 weeks of gestation).

^c^N/A: not applicable.

^d^T2: immediately after the intervention (about 20-28 weeks of gestation).

^e^T3: before birth (36-37 weeks of gestation).

^f^T4: 6 weeks post partum.

^g^T5: 3 months post partum.

^h^T6: 6 months post partum.

### Infant Neuropsychological Development

Table 3 shows a comparison of the differences in infant neuropsychological development between the digital GSH-MBI and control groups, with infant sex, maternal age, education, and average monthly household income included as covariates in the analysis. In terms of infant temperament at 6 weeks, the scores in the digital GSH-MBI group for quality of mood (β=.25, 95% CI 0.09-0.41; *P*=.002), attention span (β=.17, 95% CI 0.01-0.33; *P*=.04), distractibility (β=.30, 95% CI 0.15-0.45; *P<*.001), and adaptability (β=.22, 95% CI 0.06-0.38; *P*=.006) were lower than those in the control group. Compared with the control group, infants in the digital GSH-MBI group had less negative mood and were more likely to persist, less likely to be distracted, and more likely to be adaptive. Small to medium effect sizes were obtained for the 4 temperament types (Cohen *d*=0.35-0.66). The between-group difference in the attention span was not significant after adjustment owing to multiple tests. There were no significant differences in infant temperament at 6 months of age between the groups.

In terms of infant developmental behaviors at 6 weeks and 6 months at post partum, only problem-solving at 6 months was different between the digital GSH-MBI and control groups (β=−0.21, 95% CI −0.38 to −0.04; *P*=.02), with a small effect size. However, the *P* value was not significant after adjustment owing to multiple tests.

**Table 3:** Differences in infant neuropsychological development between the intervention and control groups.^a^

				Digital GSH-MBI^b^ group, mean (SD)		Control group, mean (SD)		β (95% CI)		Cohen *d* (95% CI)		*P* value
**Temperament**												
	**6 weeks**											
		Activity level	3.51 (0.63)		3.60 (0.65)		.07 (−0.10 to 0.23)		0.15 (−0.18 to 0.47)		.43	
		Rhythmicity	3.23 (0.61)		3.37 (0.62)		.11 (−0.06 to 0.27)		0.23 (−0.10 to 0.55)		.20	
		Approach	2.50 (0.82)		2.56 (0.92)		.03 (−0.13 to 0.20)		0.07 (−0.26 to 0.39)		.70	
		Adaptability^c^	2.57 (0.75)		2.94 (0.78)		.22 (0.06 to 0.38)		0.48 (0.15 to 0.81)		.006	
		Intensity of reaction	3.34 (0.79)		3.55 (0.88)		.12 (−0.04 to 0.29)		0.25 (−0.08 to 0.57)		.13	
		Quality of mood^c^	3.09 (0.52)		3.41 (0.68)		.25 (0.09 to 0.41)		0.53 (0.20 to 0.86)		.002	
		Attention span	3.34 (0.81)		3.61 (0.73)		.17 (0.01 to 0.33)		0.35 (0.03 to 0.67)		.04	
		Distractibility^c^	2.42 (0.71)		2.88 (0.71)		.30 (0.15 to 0.45)		0.66 (0.33 to 0.99)		＜.001	
		Threshold of reaction	4.10 (0.72)		3.94 (0.72)		−0.10 (−0.25 to 0.07)		0.21 (−0.11 to 0.54)		.25	
	**6 months**											
		Surgency	5.03 (0.96)		4.74 (1.01)		−0.16 (−0.32 to 0.01)		0.29 (−0.05 to 0.63)		.07	
		Negative affect	3.82 (1.20)		4.00 (1.29)		.06 (−0.11 to 0.23)		0.15 (−0.19 to 0.48)		.51	
		Effortful control	5.10 (0.85)		4.80 (1.02)		−0.16 (−0.33 to 0.003)		0.32 (−0.12 to 0.66)		.05	
**Development behaviors**												
	**6 weeks**											
		Communication	43.33 (12.33)		39.62 (15.34)		−0.13 (−0.30 to 0.05)		0.27 (−0.07 to 0.61)		.14	
		Gross motor	51.88 (11.08)		51.46 (10.85)		−0.02 (−0.20 to 0.15)		0.04 (−0.30 to 0.38)		.81	
		Fine motor	47.03 (10.34)		44.62 (11.33)		−0.13 (−0.30 to 0.04)		0.22 (−0.12 to 0.56)		.12	
		Problem-solving	48.99 (11.03)		46.54 (13.58)		−0.11 (−0.28 to 0.07)		0.20 (−0.14 to 0.54)		.21	
		Personal-social	45.43 (11.59)		44.08 (11.62)		−0.06 (−0.23 to 0.12)		0.12 (−0.22 to 0.46)		.51	
	**6 months**											
		Communication	51.45 (8.79)		49.35 (11.18)		−0.10 (−0.27 to 0.07)		0.21 (−0.13 to 0.55)		.26	
		Gross motor	44.78 (12.59)		42.02 (15.03)		−0.10 (−0.27 to 0.07)		0.20 (−0.14 to 0.54)		.26	
		Fine motor	48.99 (12.38)		46.13 (13.56)		−0.11 (−0.29 to 0.06)		0.22 (−0.12 to 0.56)		.20	
		Problem-solving	52.25 (9.95)		47.34 (13.42)		−0.21 (−0.38 to −0.04)		0.41 (0.06 to 0.76)		.02	
		Personal-social	45.94 (12.65)		47.77 (16.55)		−0.14 (−0.32 to 0.03)		0.28 (−0.07 to 0.62)		.11	

^a^Infant sex, maternal age, education, and average monthly household income were adjusted for.

^b^ GSH-MBI: guided self-help mindfulness-based intervention.

^c^After the adjustment of false discovery rate, their relationship was still significant.

### The Mediating Analysis of Maternal Psychological Distress Between Treatment Allocation and Infant Neuropsychological Development

The scores for the 3 types of temperament at 6 weeks (quality of mood, distractibility, and adaptability) in the digital GSH-MBI group were significantly lower than those in the control group. Thus, we used partial correlation analysis to explore the relationship between quality of mood, distractibility, and adaptability and maternal psychological distress at T2 or T3, adjusting for maternal psychological distress at T1. As shown in Multimedia Appendix 3, infants’ quality of mood was positively associated with maternal depression symptoms at T2 (*r*=.23; *P*=.01) and T3 (*r*=0.17; *P*=.047), anxiety symptoms at T2 (*r*=0.22; *P*=.01) and T3 (*r*=0.20; *P*=.02), and pregnancy-related anxiety symptoms at T2 (*r*=0.27; *P*=.001) and T3 (*r*=0.19; *P*=.02). Infants’ distractibility was positively associated with maternal depression symptoms at T3 (*r*=0.25; *P*=.003) and anxiety symptoms at T3 (*r*=0.24; *P*=.005). Infant adaptability was positively associated with maternal anxiety symptoms at T3 (*r*=0.19; *P*=.02).

**Figure 4:**
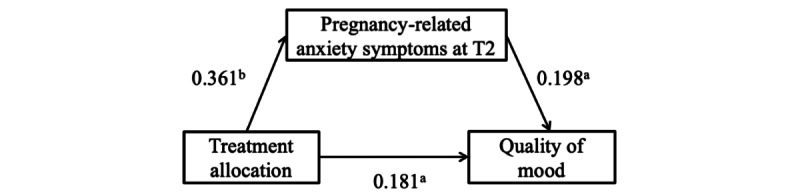
Mediation model of maternal pregnancy-related anxiety symptoms at T2 between treatment allocation and infant quality of mood. Standardized estimates are shown for relationships among treatment allocation, maternal pregnancy-related anxiety symptoms at T2, and infant quality of mood. T2: immediately after the intervention; ^a^*P*<.05; ^b^*P*<.001; Pregnancy-related anxiety at T1 (baseline, 12-20 weeks of gestation), infant sex, maternal age, education, and average monthly household income were adjusted for.

On the basis of the partial correlation analysis, we tested a total of 9 mediation models, including: the indirect effect of treatment allocation, through maternal depression symptoms at T2 or T3, to infant quality of mood; the indirect effect of treatment allocation, through maternal anxiety symptoms at T2 or T3, to infant quality of mood; the indirect effect of treatment allocation, through maternal pregnancy-related anxiety symptoms at T2 or T3, to infant quality of mood; the indirect effect of treatment allocation, through maternal depression or anxiety symptoms at T3, to infant distractibility; and the indirect effect of treatment allocation, through maternal anxiety symptoms at T3, to infant adaptability. The results showed that only one indirect effect (treatment allocation, through maternal pregnancy-related anxiety symptoms at T2, on infant quality of mood) was statistically significant. The standardized indirect effect of maternal pregnancy-related anxiety symptoms at T2 was 0.071 (95% CI 0.001-0.167; *P*=.045; Figure 4).

### Adherence

The digital GSH-MBI group completed an average of 5 (SD 2.16) weeks of modules. Of the 80 participants, 65 participants completed the intervention (intervention completion rate=81.3%).

## Discussion

### Principal Findings

To our knowledge, this is the first RCT to examine the effectiveness of a digital GSH-MBI based on a WeChat mini program for alleviating maternal psychological distress and promoting infant neuropsychological development. Overall, the results imply that pregnant women who received digital GSH-MBI displayed less psychological distress (depression, anxiety, and pregnancy-related anxiety symptoms) compared with women in the control group at all postintervention time points. In addition, the digital GSH-MBI had a ripple effect on infant neuropsychological development, and the ripple effect on infants’ quality of mood at 6 weeks was mediated by mitigating pregnancy-related anxiety symptoms.

We explored new possibilities for delivering psychological interventions to improve maternal and child health. We used the WeChat mini program as the delivery medium and included video- and audio-based dissemination of educational information and exercises. The digital GSH-MBI, based on a WeChat mini program, has several strengths for mental health care. First, the high flexibility of the digital GSH-MBI allows women to attend classes and practice mindfulness at any time and place. Second, access to the intervention is increased. The shortage of mental health services is a major challenge in mental health care in low- and middle-income countries [39]. For example, there is an estimated shortage of 40,000 psychologists in China [40]. Access to mental health care in rural and remote communities is severely low, as trained mental health professionals are concentrated in psychiatric hospitals in urban areas [40]. By delivering psychological interventions through WeChat, women in rural areas can receive the support they need. Third, costs are reduced, with fewer financial and human resources required compared with face-to-face interventions [15]. Notably, although low adherence was a limitation of web-based self-help interventions in a prior study [18], our digital GSH-MBI had high adherence and showed significant effects on maternal psychological distress, indicating that digital GSH can become a promising intervention for maternal and infant health in the future.

In this study, we noted the strongest treatment effects in 3 types of infant temperament at 6 weeks of age: quality of mood, distractibility, and adaptability. Higher scores of quality of mood represent a more negative mood, which is strongly associated with a neurotic personality in adults, both conceptually and empirically [41]. Observational studies have found that negative mood traits increase the likelihood of future emotional issues, such as internalizing and externalizing problems [42]. Our RCT is the first study to reveal that a digital GSH-MBI during pregnancy has a ripple effect on infants’ quality of mood by alleviating maternal psychological distress. This strongly supports the Developmental Origins of Health and Disease hypothesis, suggesting that prenatal exposure to environmental adversity such as maternal psychological distress during pregnancy may “program” infant neurodevelopment.

Notably, the digital GSH-MBI played a role in infants’ quality of mood through mitigating pregnancy-related anxiety symptoms rather than anxiety and depression symptoms. This finding is consistent with existing studies, which showed that pregnancy-related anxiety is a stronger predictor of offspring’s developmental outcomes than general negative emotions experienced by the mother [43,44]. These results suggest that clinicians should pay more attention to pregnant women’s psychological distress related to pregnancy and childbirth and provide the help and support they need to prevent pregnancy-related psychological distress. In addition, our findings showed that the digital GSH-MBI played a role in infant outcomes by alleviating symptoms of pregnancy-related anxiety immediately after the intervention versus before delivery. This is likely attributable to the short time interval between delivery and 6 weeks post partum. However, the immediate effect after the completion of the intervention can indirectly promote the development of newborns, and this emphasizes the important role of digital GSH-MBI in offspring development. In addition, the mediation effect was partial and incomplete, indicating that digital GSH-MBI has a direct effect on offspring outcomes, further emphasizing the unique value of digital GSH-MBI.

Distractibility is characteristic of attentional regulation [45]. Rothbart et al [46] asserted that attentional regulation in early infancy develops into an executive attention network (attentional diversion and attentional focus) in late infancy [47], which then matures during late adolescence [48]. Adaptability also reflects infant regulation skills, which are associated with future psychopathologies such as attention-deficit/hyperactivity disorder [49]. However, we did not find a mediating role of maternal psychological distress during pregnancy in the effect of the digital GSH-MBI on infants’ regulatory ability. One possible explanation is that the ripple effect may be completely direct or may involve other indirect pathways. Van den Heuvel et al [50] found that maternal mindfulness during pregnancy can directly affect infants’ self-regulation ability. In addition, mindfulness interventions might affect maternal parenting. A qualitative study [51] showed that women who participated in a mindfulness intervention during pregnancy continued to use mindfulness techniques in their relationships with their infants post partum. These participants shared that mindfulness helped them reflect on their experiences, deal with pain, and enhance their sense of pleasure with their infants. These adaptive interaction patterns and efficient care strategies may promote infants’ regulatory development. Future intervention studies are needed to examine whether maternal parenting and mother-infant bonding serve as mediating pathways for the effect of mindfulness on infant regulatory development. Although we did not observe specific mechanisms for the effect of our intervention on infants’ regulatory ability, the findings of the ripple effect highlight the value of digital GSH-MBIs during pregnancy for infant health outcomes, given that these features, which are associated with infants’ regulatory ability, are closely tied to future psychopathologies.

We did not find significant between-group differences in infant temperament at 6 months of age. On the one hand, in line with other intervention studies, the effectiveness of interventions might naturally decline over time [9]. On the other hand, postpartum factors (eg, parenting behaviors and maternal sensitivity) may have a gradually increasing impact on infants’ neuropsychological development. Hence, this may indicate that psychological interventions may be more effective in improving child health if they continue to be delivered in the postpartum period.

### Limitations

This study has some limitations. First, there might be a reporting bias because the infants’ neuropsychological development outcomes were reported by their mothers. Women in the intervention group may have become more emotionally literate and thus more likely to notice specific reactions in their infants. Measurement of neuropsychological development outcomes by trained study team members is necessary to obtain robust results. Thus, our results on infant outcomes are exploratory and should be interpreted with caution. Second, most participants reported a high socioeconomic status, limiting the generalizability of the findings to disadvantaged pregnant populations. Third, some confounding factors were not measured in our study, which may have affected the results of the mediation analysis. According to a concept map [52], child development is influenced by aspects of pregnancy, the child, daily care, and socioeconomic conditions such as maternal exposure to environmental pollutants during pregnancy and parent-child interactions. However, because of randomization, the confounding effects are likely minimized [53].

### Clinical and Research Implications

Despite these limitations, we used a relatively convenient and novel technology that allowed participants to easily access the intervention from WeChat on their smartphones or PCs. The digital GSH-MBI, which is accessible, convenient, inexpensive, and requires fewer professionals to operate, brings new prevention and intervention opportunities for mental health problems experienced by pregnant women, especially during the COVID-19 pandemic. We conducted the study in China, as WeChat is the most popular app among the Chinese population; however, our intervention package can be used in other apps. It is important to note that using existing widely used apps to deliver brief psychological interventions is more promising than developing new apps as intervention delivery platforms. In addition, the low dropout rates and high adherence to the intervention in this study support our viewpoint that easy access to information might increase participation and compliance with psychological interventions.

The nonsignificant ripple effect of the digital GSH-MBI on infant outcomes at 6 months of age indicates that interventions need to be delivered in the postpartum period. However, our findings of the intervention’s ripple effect on infant temperament at 6 weeks of age are also encouraging. First, women tend to be more motivated to make healthy changes during pregnancy than at any other time in their lives [54]. Second, during pregnancy, women have regular contact with health care providers, which increases the likelihood that they will be exposed to positive information about their health and comply with interventions [54,55]. It is crucial to screen high-risk groups for psychological distress during pregnancy and introduce digital GSH-MBI programs for pregnant women at risk.

## Appendix

Multimedia Appendix 1 Mindfulness program outline.

Multimedia Appendix 2 Parameter estimation results of mixed-effects model.

Multimedia Appendix 3 Correlation between maternal psychological distress during pregnancy and infant temperament.

Multimedia Appendix 4 CONSORT-eHEALTH checklist (V 1.6.1).

## Abbreviations

CONSORT: Consolidated Standards of Reporting Trials
EPDS: Edinburgh Postnatal Depression Scale
GAD-7: Generalized Anxiety Disorder 7-item
GSH: guided self-help
MBI: mindfulness-based intervention
RCT: randomized controlled trial
