# Supplementary material for: Effectiveness of Digital Guided Self-help Mindfulness Training During Pregnancy on Maternal Psychological Distress and Infant Neuropsychological Development: Randomized Controlled Trial
Source: J Med Internet Res. 2023 Feb 10;25:e41298. doi: 10.2196/41298 (PMC9960047; doi:10.2196/41298)
Supplement: Multimedia Appendix 1 [file jmir_v25i1e41298_app1.docx]

Multimedia Appendix 1. Mindfulness program outline.

| Module | Theme | Content | Homework | |
| --- | --- | --- | --- | --- |
|  |  |  | Formal exercise | Informal exercise |
| 1 | Introduction to the mindfulness | 1.1 Adverse effects of prenatal distress during pregnancy | Mindful breathing | Mindfulness in Everyday Life (Mindful Eating) |
|  |  | 1.2 What is mindfulness |  |  |
|  |  | 1.3 Eat raisins mindfully |  |  |
| 2 | Beyond automatic navigation | 2.1 Understand auto navigation mode | Body scan | Mindfulness in daily life (Mindful brushing, mindful bathing) |
|  |  | 2.2 Learn to distinguish between "mode of being" and "mode of action" |  |  |
|  |  | 2.3 Understand the causes of anxiety and depression |  |  |
|  |  | 2.4 Given examples of how to mindfully brush your teeth and take a shower |  |  |
| 3 | Become aware of the present experience | 3.1 Learn where your attention goes on autopilot | Mindful breathing | Mindful walking and 3 minutes breathing space exercises |
|  |  | 3.2 With practice, learn to be aware of the present experience |  |  |
|  |  | 3.3 Analyze the causes of unpleasant experiences and learn to disengage from unpleasant experiences with a 3-minute breathing space practice |  |  |
|  |  | 3.4 A mindful walking exercise guide |  |  |
| 4 | Identify avoidance responses and learn to accept difficulties | 4.1 Analyzing our aversive responses to unpleasant experiences | Mindful meditation | 3 minutes breathing space exercises |
|  |  | 4.2 Learn to live with difficulties and allow things to be as they are |  |  |
| 5 | Embrace happiness | 5.1 Review the techniques of previous lessons and learn how to deal with negative emotions | Body scan | Mindfulness works |
|  |  | 5.2 Master the 5 best ways to savor happiness |  |  |
|  |  | 5.3 Give an example of how mindfulness works |  |  |
| 6 | Mindfulness for childbirth | 6.1 Mindfulness deals with the emotional experience of childbirth | Mindful stretching | 3 minutes breathing space exercises |
|  |  | 6.2 A mindful stretching exercise guide |  |  |
