# Supplementary material for: Effectiveness of Digital Guided Self-help Mindfulness Training During Pregnancy on Maternal Psychological Distress and Infant Neuropsychological Development: Randomized Controlled Trial
Source: J Med Internet Res. 2023 Feb 10;25:e41298. doi: 10.2196/41298 (PMC9960047; doi:10.2196/41298)
Supplement: Multimedia Appendix 2 [file jmir_v25i1e41298_app2.docx]

Multimedia Appendix 2. Parameter estimation results of mixed-effects model.

| Psychological distress | Parameters | *β* | SE | *P* | 95%CI |
| --- | --- | --- | --- | --- | --- |
| Depression | [Group=1]✖️[Time=6] | -2.16 | 0.87 | .01 | (-3.87, -0.44) |
|  | [Group=1]✖️[Time=5] | -2.47 | 0.86 | .004 | (-4.16, -0.77) |
|  | [Group=1]✖️[Time=4] | -2.75 | 0.85 | .001 | (-4.42, -1.07) |
|  | [Group=1]✖️[Time=3] | -3.50 | 0.85 | ＜.001 | (-5.16, -1.83) |
|  | [Group=1]✖️[Time=2] | -2.09 | 0.83 | 0.01 | (-3.72, -0.45) |
| Anxiety | [Group=1]✖️[Time=6] | -1.75 | 0.63 | 0.01 | (-2.99, -0.51) |
|  | [Group=1]✖️[Time=5] | -1.26 | 0.63 | 0.04 | (-2.49, -0.04) |
|  | [Group=1]✖️[Time=4] | -2.48 | 0.62 | ＜.001 | (-3.70, -1.27) |
|  | [Group=1]✖️[Time=3] | -2.56 | 0.62 | ＜.001 | (-3.77, -1.35) |
|  | [Group=1]✖️[Time=2] | -2.21 | 0.60 | ＜.001 | (-3.40, -1.02) |
| Pregnancy-related anxiety | [Group=1]✖️[Time=3] | -5.65 | 0.77 | ＜.001 | (-7.17, -4.12) |
|  | [Group=1]✖️[Time=2] | -4.44 | 0.76 | ＜.001 | (-5.93, -2.94) |

Note：group=1 is digital guided self-help-mindfulness-based intervention group; and the reference group is control group.

Time=2 is immediately after the intervention, Time=3 is before birth, Time=4 is 6 weeks postpartum, Time=5 is 3 months postpartum, Time=6 is 6 months postpartum, and the reference time is the baseline.
