# Supplementary material for: Effectiveness of Digital Guided Self-help Mindfulness Training During Pregnancy on Maternal Psychological Distress and Infant Neuropsychological Development: Randomized Controlled Trial
Source: J Med Internet Res. 2023 Feb 10;25:e41298. doi: 10.2196/41298 (PMC9960047; doi:10.2196/41298)
Supplement: Multimedia Appendix 3 [file jmir_v25i1e41298_app3.docx]

Multimedia Appendix 3. Correlation between maternal psychological distress during pregnancy and infant temperament.

|  | Adaptability | Quality of mood | Distractibility |
| --- | --- | --- | --- |
| Depression at T2^c^ | 0.15 | 0.23^a^ | 0.13 |
| Depression at T3^c^ | 0.16 | 0.17^a^ | 0.25^b^ |
| Anxiety at T2^d^ | 0.14 | 0.22^b^ | 0.14 |
| Anxiety at T3^d^ | 0.19^a^ | 0.20^a^ | 0.24^b^ |
| Pregnancy-related anxiety at T2^e^ | 0.08 | 0.27^b^ | 0.12 |
| Pregnancy-related anxiety at T3^e^ | 0.05 | 0.19^a^ | 0.16 |

Note：T2: Immediately after the intervention, T3: Before delivery

^a^*P*＜.05, ^b^*P*＜.01

^c^ Depression at baseline was adjusted for.

^d^ Anxiety at baseline was adjusted for.

^e^ Pregnancy-related anxiety at baseline was adjusted for.
